# The use of pumice amended with sand media for domestic wastewater treatment in vertical flow constructed wetlands planted with lemongrass (*Cymbopogon citratus*)

**DOI:** 10.1016/j.heliyon.2021.e07423

**Published:** 2021-06-29

**Authors:** Philiphi de Rozari, Denik Sri Krisnayanti, Refli, Krispianus V. Yordanis, Maria Ratu Rosari Atie

**Affiliations:** a Department of Chemistry, Faculty of Science and Engineering, Nusa Cendana University, Kupang, Jalan Adisucipto Penfui Kupang, Indonesia; b Department of Environmental Science Post Graduate Study, Nusa Cendana University, Kupang, Jalan Adisucipto Penfui Kupang, Indonesia; c Department of Civil Engineering, Faculty of Science and Engineering, Nusa Cendana University, Kupang, Adisucipto Penfui Kupang, Indonesia; d Department of Biology, Faculty of Science and Engineering, Nusa Cendana University, Kupang, Adisucipto Penfui Kupang, Indonesia

**Keywords:** Constructed wetlands, Pumice, Lemongrass, Nutrients, Organic matter, Total coliform

## Abstract

The performance efficiency in constructed wetlands (CWs) technology is primarily affected by the media material and the types of plants used. Recently, investigations into the usage of local materials and plants in CWs has increased. Pumice is a material which is potential used as a media. However, research on amendment of pumice with other media in CWs is still limited. Therefore, this study aims to evaluate the potential of pumice amended with sand media and planted with lemongrass (*Cymbopogon citratus*) in CWs to remove organic matter, suspended solids, nutrients, and coliform. The adsorbents were characterized using X-ray diffraction, FTIR and XRF followed by adsorption experiments for PO_4_–P. Furthermore, Six vertical flow (VF) mesocosms with a diameter of 10.2cm and 55cm depth were established over six months. The treatments were based on percentage of sand media amended with pumice and planted with lemongrass. Furthermore, the barren media were applied to investigate the effect of lemongrass. The loading rate of domestic wastewater into the VF mesocosms was 2 L/day while inflows and outflows were determined for nutrients, organic matter, suspended solids and coliform. The adsorption of PO_4_–P followed the Langmuir model with adsorption capacity was 0.089 and 0.067 mol/g for pumice and sand, respectively. The results also showed that the removal efficiency of TSS, COD, NO_3_–N, NO_2_–N, PO_4_–P and total coliforms were in the range of 93.7–97.3 %, 52–83 %, 63–86 %, 51–74%, 81–88 % and 92–97 %, respectively. Based on the results, the highest removal efficiency was observed in the sand media amended with 50 % pumice and planted with lemongrass, while the lowest was found in the barren sand media.

## 1 Introduction

The East Nusa Tenggara Province (ENTP) is an area in Indonesia dominated by archipelagic drylands with an annual rainfall of 600–2700mm BPS-NTT (2020). This province is located in tropical climate and experiences a long dry–season, meaning that water availability is a major challenge. Moreover, the rising population of this area has led to an increase in water consumption and wastewater production. Majority of the wastewater is discharged to the environment without any proper treatment, leading to public health problems and environmental pollution. This condition is exacerbated by the absence of adequate wastewater treatment plants due to high investment and operational costs.

Constructed wetlands (CWs) are promising wastewater treatment alternatives due to the numerous ecological benefits, low operational and maintenance costs as well as low energy requirements (Kadlec and Wallace, 2008; Kumar and Dutta, 2019). This technology is potentially used for treating wastewater in tropical areas due to the abundant biodiversity, warm temperature and high humidity (Nakase et al., 2019; De Rozari et al., 2020a, De Rozari et al., 2020b; Konnerup et al., 2009). Inside the CWs, the pollutants are removed by physical, chemical and biological treatments such as sedimentation, volatilization, adsorption, microbial decomposition, and plant uptake (Kadlec and Wallace, 2008; Gupta et al., 2015). Several studies have reported that media and plants are the key elements in CWs systems for removing nitrogen, phosphorus, organic matter, and bacteria from domestic wastewater (Ilyas and Masih, 2017; Yang et al., 2018; Vohla et al., 2011).

The use of *Phragmites, Typha and Cyperus* is common in CWs, particularly in tropical regions (Casierra-Martínez et al., 2017; Weerakoon et al., 2016). However, these species are not considered as indigenous plants and might be classified as invasive plants (Kulmatiski et al., 2011). Recently, research on suitability of local media and plants in tropical areas for pollutant removal using CWs has generated massive interest. The use of local materials and plants in abundant amounts potentially reduce construction and operational costs. Therefore, identifying local materials and plants to efficiently treat wastewater for removing pollutants is critical. Sandoval-Herazo et al. (2018) showed that tropical ornamental plants (*Spathiphyllum wallisii* and *Zantedeschia aethiopica*) removed BOD (57–74%), PO_4_–P (34–48%), NO_3_–N (40–59%), and coliforms (62–65%). Meanwhile, Zurita et al. (2009) designed CW experiments using volcanic red-orange extrusive gravel planted with *Zantedeschia aethiopica*, *Strelitzia reginae*, *Anturium andreanum* as well as *Agapanthus africanus* and reported that the average removals of BOD, org-N, NH_4_–N, TP and coliforms were 80%, 51%, 72%, 50% and 97%, respectively. Pincam et al. (2020) also reported that *Thalia geniculata* and *Cyperus involucratus Rottb*. planted in tropical areas served as a good alternative for phosphorus and nitrogen removal particularly in a stressful condition. Furthermore, Yang et al. (2007) reported that lemongrass (a perennial grass and related to vetiver grass) could potentially tolerate inundation and uptake and store nutrients from wastewater. In addition, lemongrass is capable of removing suspended solids due to high root density and spreading growth pattern (Wanyama et al., 2012). These findings suggest that indigenous plants potentially enhance pollutant removal in CW systems.

The type of material used as a media is another important factor for successful wetland treatment process. Vohla et al. (2011) classified the materials for CWs into three categories namely natural materials, man-made products as well as by-products. Sand and gravel are widely used in CW systems due to low cost. However, these materials are not ideal in removing pollutants particularly phosphorus and nitrogen (Yang et al., 2018). Pumice is a type of volcanic rock material formed by solidified processes of volcanic lava. This material has low weight, high porosity and large surface area. Besides, the skeleton structure of pumice builds open channels which allow water and ions to travel in and out of the crystal structure (Liu et al., 2014). Pumice mainly consists of over 50% of silica (SiO_2_), while silica surface contains silanol groups which potentially interact with polar organic compounds, other functional groups and cations (Heibati et al., 2014).

As a low cost material, pumice has been used as a potential alternative material for pollutant removal from wastewater. Using the laboratory scale, Cheng et al. (2018) examined the use of pumice-woodchip packed stormwater biofilter (PWSWBF) for pollutant removals from livestock farm stormwater. The results showed that the PWSWBF had high removal efficiency for TCOD (95%), TN (70%), NH_4_–N (86%), NO_3_–N (100%) and TP (100%), indicating that the presence of pumice significantly affect the removal of pollutants. Furthermore, Niu et al. (2020) compared the removal efficiency of TSS, TCOD, TN and TP in CWs loaded with road stormwater based on variation of pumice and woodchip. It was reported that the increase of TSS, TCOD TP removal efficiency was in line with the increase of pumice. These two reports were focused on stormwater. In addition, batch experiment conducted by Fetene and Addis (2020) reported that pumice removed 95% phosphate from secondary treatment of municipal wastewater. Çifçi and Meriç (2016) evaluated the application of pumice for treated wastewater and concluded that adsorption mechanisms play an important role for pollutant removal. Therefore, augmenting sand media with pumice in CWs might be prospective to improve the removal efficiency of pollutants. To the best of authors’ knowledge, literatures focused on the effect of pumice amended with sand media planted with local plant to remove pollutants from domestic wastewater are limited. Therefore, this study aims to evaluate the potential of sand media amended with pumice and planted with lemongrass (*Cymbopogon citratus*) in CWs for removal of nutrients, organic matter, suspended solids and coliform.

## 2 Methods/materials

### 2.1 Media characterization

The sand was sourced from the river and sampled in Takari Kupang District while pumice was collected from Tuamese Village, Biboki Anleu Subdistrict, the District of North Centre Timor (Timor Tengah Utara). To obtain baseline information, sand and pumice were characterized based on physicochemical aspects. The parameters used for media characterization include porosity, organic matter, pH, cation exchange capacity (CEC), crystal structure (XRD), chemical composition of materials (XRF), and functional groups (FTIR). Furthermore, characterization of pH was determined according to Methods of Analysis for Soils of Arid and Semi-Arid Regions (Bashour and Sayegh, 2007), while organic matters were determined using gravimetric methods based on ASTM D2974. Moreover, CEC was determined using silver thiourea methods (Pleysier and Juo, 1980). Detailed description of media characterization has been reported in previous paper De Rozari et al., 2020a, De Rozari et al., 2020b.

### 2.2 Adsorption study

In this study, sand and pumice (diameter 1–2 mm) were used. Prior to the isoterm adsorption study, the materials were washed with deionised water and dried at 80 °C for 24 h. Afterwards, the isotherm adsorption was carried out by weighing 0.5g of each adsorbent and the weighed samples were placed in the containers. Each container was added with 20ml PO_4_–P solution with various concentrations (0.00; 0.01; 0.02; 0.04; 0.06; 0.08; 0.1; 0.2; 0.4; 0.6; 0.8; 1; 2; 5; 10; 20; 40; 60; 80; 100 mg/L). The containers were shaken in mechanical shaker for 8 h at 350 rpm. These processes were conducted in triplicates and the filtrates were collected in the containers and analyzed for PO_4_–P using molybdenum blue methods with spectrophotometry according to standard methods for wastewater examination (APHA, 2005). The data were analyzed using Langmuir adsorption isotherm with assumption monolayer adsorption sites. The formula of Langmuir isotherm is presented below (McBride, 2000):

(1) $$q=\frac{bKC}{1+KC}$$

Where q is the quantity of PO_4_–P adsorbed per gram of media (mg/g), C is the equilibrium concentration of PO_4_–P (g/m3), K (L/mg) represents the equilibrium constant, b (mg/L) is the adsorption capacity. The linear form of Langmuir formula is presented below:

(2) $$\frac{C}{q}=\frac{1}{bK}+\frac{C}{b}$$

Furthermore, the adsorption energy (ΔG^0^) is obtained using thermodynamic formula (Huang et al., 2015)

(3) $$E_{Ads}=-\text{Δ}G_{0}=RT\;\ln\;K$$

Where R is the gas constant, T is the temperature in Kelvin and K is thermodynamic equilibrium constant.

### 2.3 Experimental design

The experiment was carried out from February to June 2019 at the Department of Chemistry, Nusa Cendana University, Kupang ENTP. Meanwhile, the temperature in 2019 ranged from 21.7 to 33.5 °C while the relative humidity ranged from 70–87 %. In Kupang ENTP, there are eight months in the dry season (March–October) and only four months in wet season (November–February). The precipitation during wet season ranged from 179–499 mm/month (BPS-Kota-Kupang, 2019).

**Figure 1:**
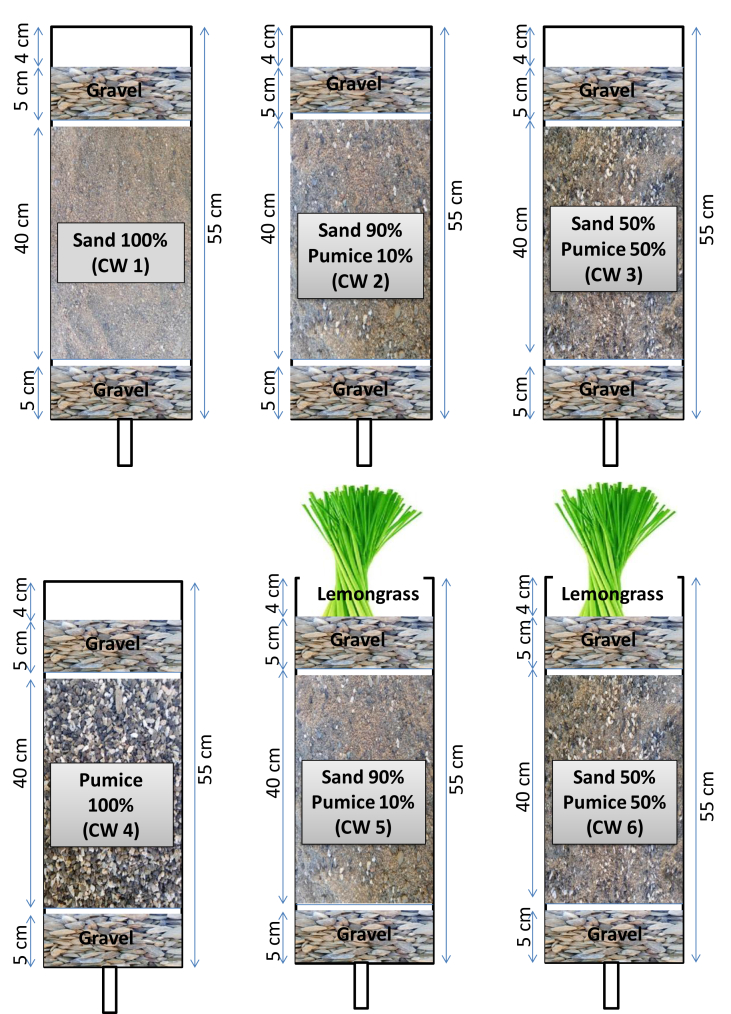
Schematic diagram of column cells.

**Figure 2:**
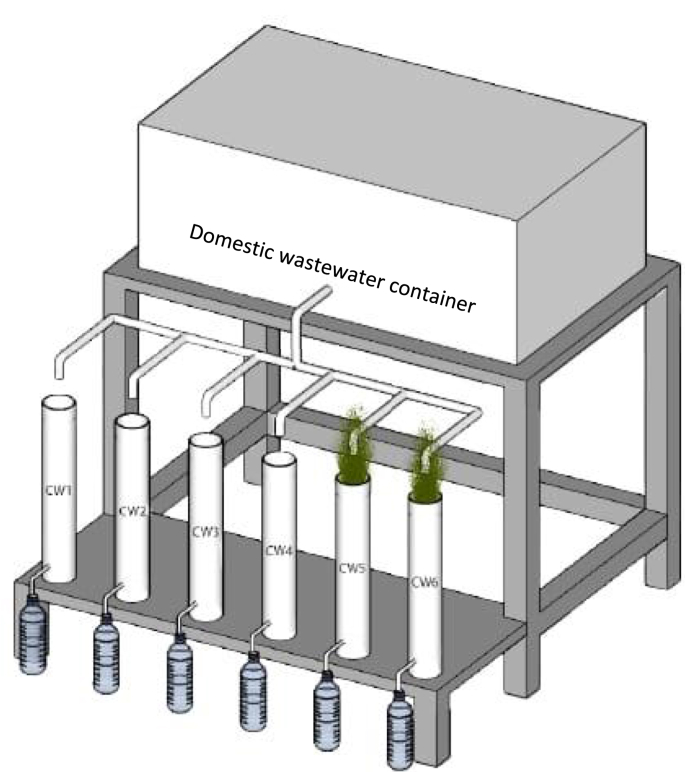
Schematic layout of VF mesocosms setup.

Six treatments were prepared using sand media amended with different percentages of pumice content, with and without vegetation as presented in Figure 1. The vertical flow (VF) mesocosms were made from polyvinyl pipe column with 10.2 cm inner diameter and 55cm depth. The mesocosms were introduced with 5cm of gravel at the bottom (diameter 10–20mm) followed by 40cm of media in the middle (diameter 2–4mm), and 5cm of gravel (diameter 10–20mm) at the top with a total depth of 50cm. More detailed experimental design has been presented in the previous paper (De Rozari et al., 2020a, De Rozari et al., 2020b). There were four barren VF mesocosms (CW1, CW2, CW3 and CW4) and two VF mesocosms (CW5 and CW6) planted with lemongrass (*Cymbopogon citratus*). The barren VF mesocosms (CW2 and CW3) were used to investigate the influence of lemongrass in removing pollutants. Meanwhile, the domestic wastewater was stored in 50 L containers and the inflows were passed to the six VF mesocosms with a continuous flow and hydraulic loading rate (HLR) of 2 L/day corresponding to hydraulic retention time (HRT) of 1 day. Furthermore, the wastewater was topped up with new inflows every three days. A schematic pilot scale of mesocosms setup with and without lemongrass is illustrated in Figure 2.

### 2.4 Water sample and collection

Water samples were collected from the inflows and outflows at the end of each week for the first five weeks starting from February to April 2019 and then every two weeks from April to August 2019. The parameters measured in the field experiments include pH, using pH meter (CX-401 Elmetron, Witosa Poland), based on the standard methods for the examination of water and wastewater (APHA, 2005). Furthermore, the parameters analyzed in the laboratory include chemical oxygen demand (COD), total suspended solids (TSS), total coliforms, nitrate (NO_3_–N), nitrite (NO_2_–N) and phosphorus (PO_4_–P). All measurements were triplicated while COD, TSS and total coliforms were analyzed immediately after sampling. Meanwhile, the water samples used for nutrient analysis (N and P) were directly stored in a cool box at 4 °C for transport and then frozen until analyzed. All the analyses were conducted based on the standard methods for the examination of water and wastewater (APHA, 2005).

### 2.5 Data analysis

Data obtained from water measurements were analyzed using SPSS 21. Meanwhile, the mean and standard deviation of inflow and outflow concentrations of chemical oxygen demand (COD), total suspended solids (TSS), total coliforms, nitrate (NO_3_–N), nitrite (NO_2_–N) and phosphorus (PO_4_–P) were also determined. Furthermore, the percentage removal efficiency was calculated to determine the performance of each VF treatment while the percentage removal efficiency was calculated using the following equation.

(4) $$\text{Removal}\;\left(\%\right)=\frac{C_{in}-C_{ef}}{C_{in}}x100\%$$

where C_in_ and C_ef_ are inflow and outflow of chemical oxygen demand (COD), total suspended solids (TSS), total coliforms, nitrate, nitrite and phosphorus concentrations. The one-way ANOVA analysis was carried out to compare the differences among treatments for each parameter. Moreover, the Tukey HSD post-hoc tests with the level of α < 0.05 was performed to investigate the significant difference of each treatment.

## 3 Result and discussion

The results are classified into three sections, namely media characterization, isoterm adsorption studies of media and performance of six treatments for removing suspended solids, COD, NO_3_–N, NO_2_–N, PO_4_–P and total coliform.

### 3.1 Media characterization

The media plays a crucial role in pollutant removal, hence, the understanding of media characterization is important. Meanwhile, media characterization using XRD, XRF and FTIR has been reported in previous paper (De Rozari et al., 2020a, De Rozari et al., 2020b). Table 1 shows the summary of media characterization.

**Table 1:** Physical and chemical characteristics of adsorbents.

Chemical Composition	Media	
	Sand	Pumice
SiO_2_ (%)	38.1	50
Fe_2_O_3_ (%)	14.9	16.1
CaO (%)	41.9	11.8
Al_2_O_3_ (%)	-	10
K_2_O (%)	1.24	8.42
TiO_2_ (%)	1.55	1.63
MnO (%)	1.78	0.63
BaO (%)	0.10	0.45
ZrO_2_ (%)	0.02	0.14
CuO (%)	0.09	0.11
V_2_O_5_ (%)	0.05	0.05
ZnO (%)	0.02	0.048
Others	0.25	0.62
Physicochemical parameters		
Porosity (%)	35	61
pH	8.05 ± 0.02	8.17 ± 0.02
Cation exchange capacity cmol (+)/kg	5.89 ± 0.59	6.17 ± 0.30

**Figure 3:**
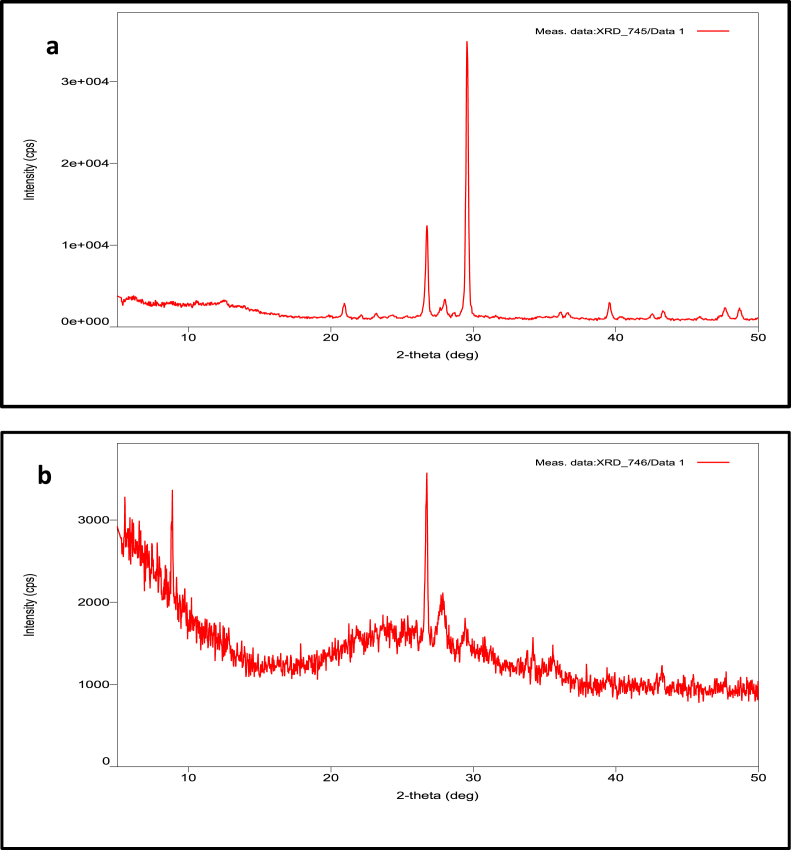
XRD Spectra of (a) sand media and (b) pumice.

Based on the results, SiO_2_ had 50% in pumice and 38% in sand media. Besides, the presence of SiO_2_ is supported by the FTIR spectra which indicated that both pumice and sand mainly contain siloxane and silicon functional groups (De Rozari et al., 2020a, De Rozari et al., 2020b). These functional groups play an important role in adsorption mechanism. In addition, both sand and pumice contain cations such as Al^3+^_,_ K^+^, Ca^2+^, Fe^3+^, Mn^2+^, Zn^2+^ and Ba^2+^. These cations play a crucial role in ion-exchange mechanisms. In general, the chemical components observed in this study are similar with other pumice and sand contents reported by other studies (Farizoglu et al., 2003; Çifçi and Meriç, 2016). Furthermore, Table 1 shows that pumice had higher percentage of SiO_2_, Al_2_O_3_, K_2_O, BaO, and ZnO compared to sand media, meanwhile, sand media had higher CaO, and MnO compared to pumice. The mineral structure determined by XRD analysis is presented in Figure 2. It shows that the sand media mainly consists of calcite and quartz (SiO_2_), while pumice contains quartz (SiO_2_) which is one of the main components of pumice (see Figure 3).

### 3.2 Adsorption isoterm

**Figure 4:**
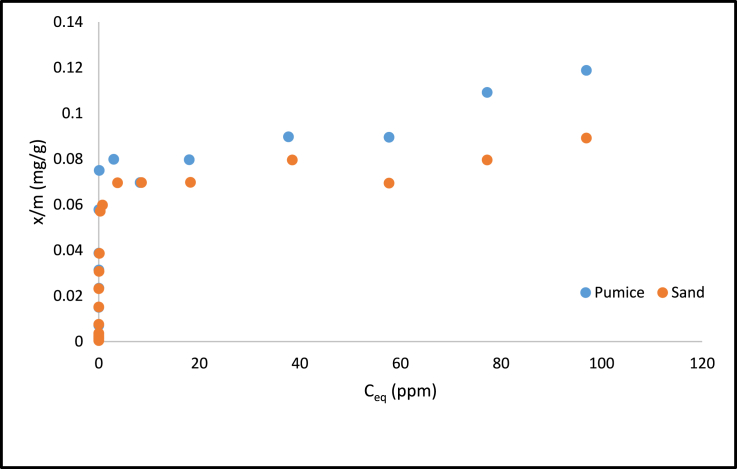
Langmuir isotherm adsorption of PO_4_–P in the sand 100% and pumice 100%.

The isotherm pattern of sand (100%) and pumice (100%) in the varied PO_4_–P concentrations is shown in Figure 4. At low concentrations, there is a significant increase in adsorption on the media. The PO_4_–P concentration began to reach equilibrium where the phosphate slowly reached a saturated state or closed to strong adsorption capacity (SAC).

**Figure 5:**
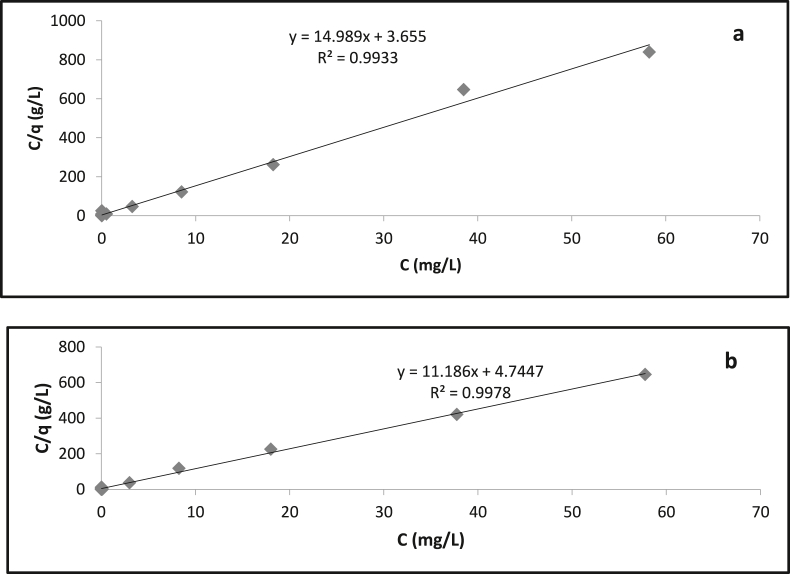
Linear model of Langmuir Isotherm adsorption from (a) sand 100% (b) pumice 100%.

Based on the linear Langmuir model, there was a strong relationship between C⁄q and C (Figure 5). This indicates that PO_4_–P adsorption isotherm in the sand had a good relationship with a regression coefficient close to 1. Moreover, the adsorption energy produced by the media ranged from 26.47 - 29.30 KJ/mol (Table 2). This was in line with Anshar et al. (2015). The results were categorized as chemisorption because adsorption energy was greater than 20 KJ/mol. Pumice had the highest adsorption capacity compared to sand which had the lowest adsorption capacity. The CaO, Fe_2_O_3_ and Al_2_O_3_ might be the main contributor for PO_4_–P adsorption in the pumice and sand adsorbent.

**Table 2:** Isotherm adsorption of sand (100%), and pumice (100%).

Adsorbent	Coefficient Correlation (R^2^)	Adsorption Capacity (b) mol/g	Equilibrium Constant (K)	Adsorption Energy (ΔG) KJ/mol
Pumice 100%	0,997	0,08944	73.024,163	27,93
Sand 100%	0,993	0,06675	126.941,694	29,30

### 3.3 Pollutant removals

This study focused on the performance of each treatment for removing suspended solids, COD, NO_3_–N, NO_2_–N, PO_4_–P and total coliform from domestic wastewater. The mean inflows were 1160 mg/L, 347 mg/L, 2.93 mg/L, 0.22 mg/L, 3.18 mg/L and 1813 CFU/100 ml, respectively. Furthermore, the results showed significant differences between inflow and outflow concentrations for all parameters (α < 0.05). The outflows and detailed performance of each treatment for all parameters over a period of six months are presented below.

#### 3.3.1 Removal of suspended solids

**Figure 6:**
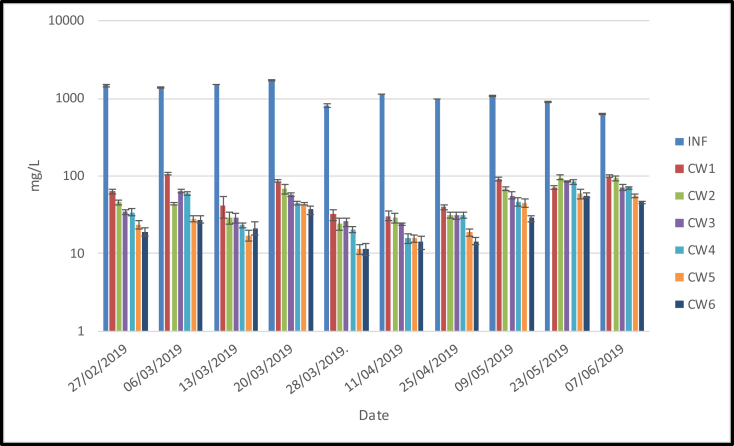
TSS (*x* ± SD) concentrations (mg/L) in six different treatments loaded with domestic wastewater.

Figure 6 shows that the inflow concentrations of TSS fluctuated, ranging from 621 to 1722 mg/L while the mean concentrations in the outflows ranged from 27.4 –66.0 mg/L. The data presented in Figure 6 shows that barren sand media (CW1) had the highest outflow concentration ranging from 30.5–106.0 mg/L while the lowest outflow concentration (11.3–56.0) was found in the sand media amended with 50% pumice and planted with lemongrass (CW6). The removal efficiency of TSS ranged from 93.7 (CW1)–97.3 (CW6) %. These results were in line with other studies that obtained removal efficiency above 90% (Leiva et al., 2018; López et al., 2015; Qomariyah et al., 2017).

Furthermore, Figure 6 also shows that TSS concentrations in the outflows reduced with the addition of pumice in the sand media (CW1–CW4). However, the one-way ANOVA analysis (α < 0.05) showed no significant differences among CW1–CW4, which confirmed that the addition of pumice did not affect TSS removal. Concerning the effect of vegetation on CWs, the data show that the mean outflow concentrations of planted media ranged from 27.4 (CW6) to 31.7 (CW5) mg/L. In contrast, the unplanted media had a mean outflow concentration ranging from 47.6 (CW3) to 52.8 (CW2) mg/L. Although the trend showed reducing outflow concentrations between barren (CW2 and CW3) and planted media (CW5 and CW6), there were no significant differences in TSS between both media. Several studies reported no significant differences between barren and planted media in the suspended solid removal experiments (Gupta et al., 2015; Haddis et al., 2020; Gikas and Tsihrintzis, 2012). This indicates that the presence of lemongrass did not significantly reduce TSS concentrations in the outflows. Hence, the main factor influencing TSS removal is probably the hydraulic retention time (HRT) and the physico-chemical processes. Microbial degradation of organic suspended solids might have slower mechanisms in TSS removal compared to physico-chemical processes.

As shown in Figure 6, only the outflow concentration of sand media (50%) and pumice (50%) planted with lemongrass (CW6) fulfilled the standard (<30 mg/L) for wastewater discharge guidelines according to the Indonesian Ministry of Environmental Regulation Number P.68/Menlhk/Setjen/Kum.1/8/2016. This result indicates that the sand media (50%) and pumice (50%) planted with lemongrass (CW6) is useful as an alternative wastewater treatment to solve water challenges in semi arid regions.

#### 3.3.2 Removal of chemical oxygen demand (COD)

**Figure 7:**
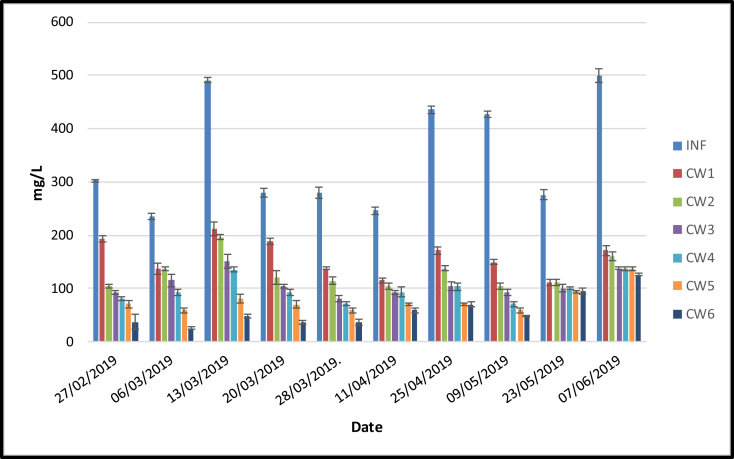
COD (*x* ± SD) concentrations (mg/L) in six different treatments loaded with domestic wastewater.

Figure 7 shows that the inflow concentrations of COD irrigated with domestic wastewater ranged from 235 to 499 mg/L. The mean concentrations of COD in the outflows ranged from 58 (CW6) to 158 (CW1) mg/L (Figure 7), which corresponded to removal efficiency ranging from 52 (CW1) to 83% (CW6). Based on the results, the increase percentage of pumice in the media reduced the COD outflow concentrations. However, the one-way ANOVA analysis (α < 0.05) showed that there were no significant differences among CW1–CW4. This indicates that the addition of pumice did not influence the COD removal. In addition, significant differences in COD outflow concentrations were observed between CW2 and CW5 indicating that the presence of lemongrass influenced COD removal. Furthermore, the outflow COD concentrations showed that the media planted with lemongrass was below the standard for domestic wastewater discharge (<100 mg/L) according to the Indonesian Ministry of Environment Regulation No P.68/Menlhk/Setjen/Kum.1/8/2016.

The COD removal efficiency obtained in this study agreed with Leiva et al. (2018) which reported a COD removal efficiency above 60%. Meanwhile, this was lower compared to Gonzalo et al. (2017) which reported a 91–99 % removal efficiency using sand media loaded with municipal wastewater. The planted media results are in congruent with Zurita et al. (2009) which obtained 75–83 % COD removal using media planted with ornamental vegetation (*Zantedeschia aethiopica*, *Strelitzia reginae*, *Anturium andreanum* and *Agapanthus africanus*). Moreover, the COD reduction mechanism is affected by several factors including HRT, physico-chemical (adsorption and precipitation) as well as microbial mechanisms (Gupta et al., 2015; Trang et al., 2010).

The performance of treatments planted with lemongrass were significantly higher compared to the barren media. This result was in line with several studies which reported that the use of plants significantly improved the performance of CW for COD removal (Trang et al., 2010; Leto et al., 2013; Caselles-Osorio et al., 2017). Furthermore, Mustapha et al. (2018) reported that planted CW culminated in 20 % higher BOD removal efficiency compared to the unplanted. Meanwhile, oxygen plays an important role in organic matter removal through aerobic degradation. The presence of plants in CWs provide dissolved oxygen used by heterotrophic bacteria for organic matter degradation, delay the wastewater flowing through media, trap the particulate materials and stimulate microbial growth (Zurita et al., 2009; Saeed and Sun, 2012; Khalifa et al., 2020).

#### 3.3.3 Removal of nitrogen

**Figure 8:**
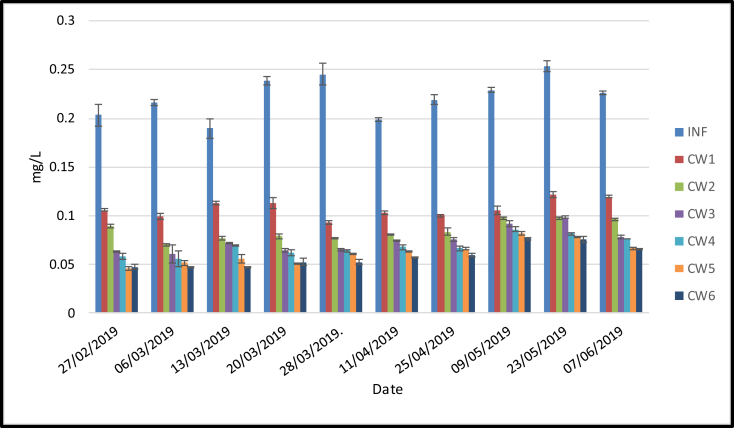
NO_2_–N (*x* ± SD) concentrations (mg/L) in six different treatments loaded with domestic wastewater.

**Figure 9:**
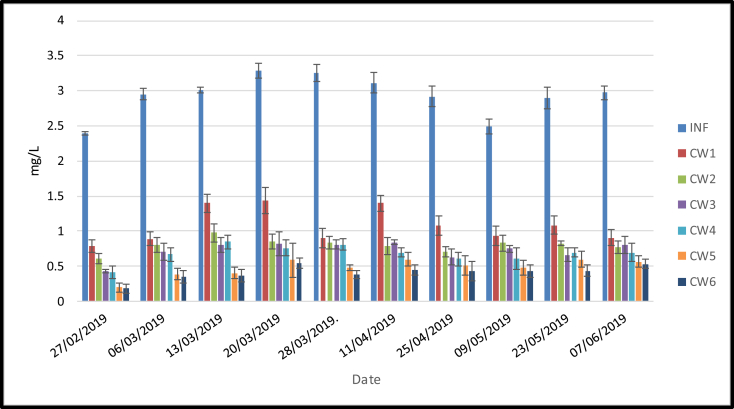
NO_3_–N (*x* ± SD) concentrations (mg/L) in six different treatments loaded with domestic wastewater.

The nitrogen parameters analyzed in this research include nitrite (NO_2_–N) and nitrate (NO_3_–N). As shown in Figures 8 and 9, the highest outflow concentrations of both parameters was found in the sand media (CW1). Meanwhile, the lowest outflow concentration was in the sand media amended with 50% of pumice and planted with lemongrass (CW6). The one-way ANOVA analysis (α < 0.05) showed significant differences in NO_2_–N and NO_3_–N outflow concentrations among the six treatments when the mesocosms were loaded with domestic wastewater (Table 3). Furthermore, the post hoc tests showed that NO_2_–N and NO_3_–N had better performance in the planted sand media amended with 50% pumice. This was confirmed by removal efficiency of NO_2_–N and NO_3_–N which ranged from 51 (CW1) to 74 (CW6) % and 63 (CW1) to 86 (CW6) %, respectively.

**Table 3:** Significant differences of NO_2_–N and NO_3_–N loaded with secondary clarified wastewater among the treatments (α < 0.05).

	CW1	CW2	CW3	CW4	CW5	CW6
CW1	-	x+	x+	x+	x+	x+
CW2	x+	-		-	x+	x+
CW3	x+	-	-	-	+	+
CW4	x+	-	-	-	-	+
CW5	x+	x+	+	-	-	-
CW6	x+	x+	x+	x	-	-

x: significant difference of NO_2_–N (α < 0.05).

+: significant difference of NO_3_–N (α < 0.05).

: no significant differences.

Several mechanisms for removal of oxidized nitrogen from subsurface CW include physico-chemical mechanism, i.e. adsorption, as well as biological mechanism, i.e. ammmonification, nitrification, denitrification, and plant uptake (Saeed and Sun, 2011). Besides, nitrogen is available in domestic wastewater either in organic or inorganic forms (Abou-Elela et al., 2013). Nakase et al. (2019) reported that at pH higher than 8, ammonium is sequestered on the media by adsorption mechanism and converted to ammonia (NH_3_) gas via ammonification. The results showed that pH in the media ranged from 8.05–8.17. Therefore, the removal of nitrogen via ammonification and adsorption mechanisms is achieved. The increase observed in the pumice content amended in the sand (CW1–CW4) media enhanced the removal efficiency of NO_2_–N and NO_3_–N. This is probably because pumice has higher cation exchange capacity (CEC), porous structure as well as specific surface area compared to the sand media. In addition, surface charge on pumice bind distinctively with microbial cells, chemical compounds and ions. This condition might lead to the adsorption and trapping of organic and inorganic compounds in the porous media structure. Based on the results, the outflow concentrations in planted media were significantly lower compared to the barren. This indicated that biological processes including nitrification, denitrification, and plant uptake play a significant role in removing NO_2_–N and NO_3_–N. In addition, the nutrients trapped in the porous media promote microbial growth leading to the removal of oxidized nitrogen. Nitrification and denitrification mechanisms tends to occur in the removal of NO_2_–N and NO_3_–N. The overall reactions are expressed in the following chemical reaction:

Nitrification (Saeed and Sun, 2012)

(5) $$2{{\text{NH}}_{4}}^{+}+4{\text{O}}_{2}\to 2{{\text{NO}}_{3}}^{-}+4{\text{H}}^{+}+2{\text{H}}_{2}\text{O}$$

Denitrification (Kadlec and Knight, 1996)

(6) $${{\text{NO}}_{3}}^{-}+1.08{\text{CH}}_{3}\text{OH}+0.24\;{\text{H}}_{2}{\text{CO}}_{3}\to 0.056\;{\text{C}}_{5}{\text{H}}_{7}{\text{NO}}_{2}+0.47{\text{N}}_{2}+1.68{\text{H}}_{2}\text{O}+{{\text{HCO}}_{3}}^{-}$$

Further investigation is needed to clearly understand the role of sand, pumice, lemongrass, and hydraulic loading rate in removing NO_2_–N and NO_3_–N. This information is important to optimize nitrogen removal efficiency.

#### 3.3.4 Removal of phosphorus

**Figure 10:**
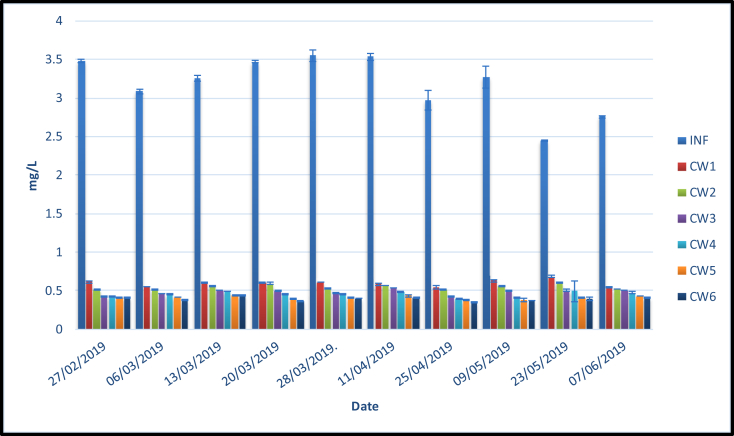
PO_4_–P (*x* ± SD) concentrations (mg/L) in six different treatments loaded with domestic wastewater.

Figure 10 shows that PO_4_–P inflows concentrations ranged from 2.4–3.6 mg/L. Meanwhile, the outflows ranged from 0.39 (CW6)–0.59 (CW1) mg/L which corresponded to a removal percentage of 88 and 81%. The highest outflow concentration was found in the pure sand media (CW1) while the lowest was found in the sand media amended with 50% pumice and planted with lemongrass. Furthermore, there were no significant differences among treatments. This indicates that the addition of pumice and planted media did not affect the PO_4_–P outflow concentrations.

This result was in line with Zhu et al. (2013) which reported that phosphorus removal efficiency ranged from 78 to 84% using a medium consisting of four layers; 10cm of gravel placed at the bottom, followed by 10 cm of cinder, 12cm of ceramsite and 6cm of sand on the top and planted with *Canna indica* L. Meanwhile, Li et al. (2013) obtained 85 % phosphorus removal efficiency using water quenched slag (WQS) media planted with *Canna indica* L when loaded with domestic sewage. Perdana et al. (2020) reported that this value reached 91% using gravel media planted with several plants. Jóźwiakowski et al. (2017) obtained a phosphorus removal efficiency of 97 % using different particle sizes with diameter 5–10mm of carbonate-silica rock media.

The phosphorus removal is often carried out using several mechanisms namely adsorption, precipitation and plant uptake. In this study, there were no significant differences between pure and sand media amended with pumice due to the low HRT and the presence of similar functional groups. Furthermore, the FTIR spectra indicated that the media surface mainly contained siloxane and silicon functional groups. Phosphate ions could be adsorbed into the siloxane surface areas. Vohla et al. (2011) noted that phosphorus removal mainly occurred via adsorption and precipitation reactions with Ca, Al, and Fe. In this study, the existence of calcium oxides and FeO in pumice and sand might lead to adsorption and precipitation reaction. Several studies revealed that planted wetland played a major role in phosphorus removal (Wu et al., 2008; Saz et al., 2018). However, this result shows no noticeable differences between barren and planted media. This suggests that plant uptake mechanisms did not play a significant role in phosphorus removal.

#### 3.3.5 Removal of coliforms

**Figure 11:**
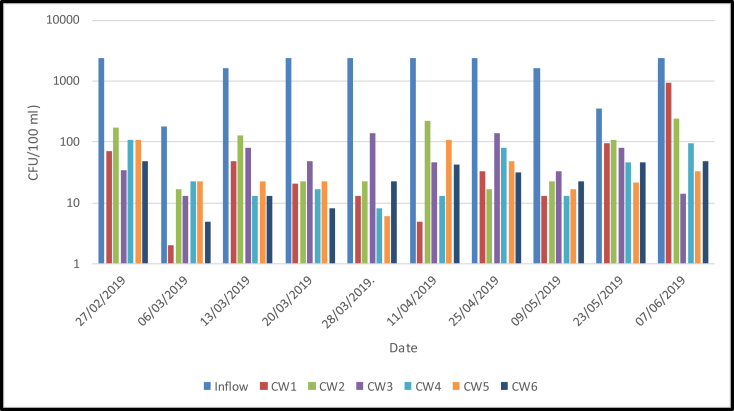
Population of total coliforms (CFU/100 ml) in six different treatments loaded with domestic wastewater.

The total coliforms in the VF mesocosms with six different media treatments is shown in Figure 11. It ranged from 180 to 2400 CFU/100 ml in the inflow, meanwhile, the mean total coliforms in the outflow reduced significantly. The total coliforms in the outflows ranged from 29 (CW6) to 122 (CW1) CFU/100 ml. Furthermore, the highest removal efficiency (97%) was found in the sand media amended with 50% pumice and planted with lemongrass (CW6), while the lowest (92%) was found in the sand media amended with 10% pumice. The total coliforms in the outflows fulfil the standard for domestic wastewater discharge (<3000 CFU/100 ml) based on the Indonesian Ministry of Environment Regulation No P.68/Menlhk/Setjen/Kum.1/8/2016.

Based on the results, there were no significant differences in the total coliform among the six treatments. The removal of total coliforms is mainly due to filtering mechanisms of the media (Gikas and Tsihrintzis, 2012; Headley et al., 2013; Vacca et al., 2005). Furthermore, Kadlec and Wallace (2008) stated that four main factors improve pathogen removal namely (1) finer bed material, (2) warmer water temperature, (3) longer hydraulic retention time (HRT), and (4) shallower bed depth. The use of plant potentially reduces HRT and enhances filtering capability, but in this study, the existence of plants did not significantly influence the results. Therefore, the effect of plants in removing pathogen rely on specific conditions.

## 4 Conclusion

Based on the results, the adsorption of PO_4_–P in pumice and sand media followed Langmuir isotherm with adsorption energy 27.47 and 29,30 KJ/mol, respectively. Thus, it can be classified as chemisorption. The removal efficiency of the six different treatments ranged from 93.7–97.3 %, 52–83 %, 63–86 %, 51–74 %, 81–88 % and 92–97 % for TSS, COD, NO_3_–N, NO_2_–N, PO_4_–P and total coliforms removal, respectively. The highest removal efficiency was found in the sand media amended with 50% pumice and planted with lemongrass while the lowest was found in the barren sand media. Furthermore, there were no significant differences among the outflow concentrations of TSS, PO_4_–P, and total coliforms. This suggests that the addition of pumice into the sand and planted media did not significantly improve the removal efficiency of the parameters. Moreover, sand media amended with 50% pumice and planted with lemongrass had significant COD, NO_3_–N, NO_2_–N removal. This indicates that plant uptake mechanisms contribute to removal efficiency. In general, to remove pollutant from wastewater, the results show that the addition of pumice into the media is less effective. However, when the media is planted with lemongrass, the removal of COD, NO_3_–N, NO_2_–N improves significantly. Therefore, more investigation is required to improve the pumice performance for media amendment in vertical flow constructed wetlands.

## Declarations

### Author contribution statement

Philiphi de Rozari: Conceived and designed the experiments; Performed the experiments; Analyzed and interpreted the data; Contributed reagents, materials, analysis tools or data; Wrote the paper.

Denik Sri Krisnayanti: Conceived and designed the experiments; Analyzed and interpreted the data; Wrote the paper.

Refli: Analyzed and interpreted the data.

Krispianus V. Yordanis & Maria Ratu Rosari Atie: Performed the experiments.

### Funding statement

This work was supported by the Directorate of Research and Community Service, Department of Strengthening Research and Development at the Ministry of Research, Technology/National Agency for Research and Innovation under contract No 127/UN15.19.1.2/SP2H/LT/2020.

### Data availability statement

Data will be made available on request.

### Declaration of interests statement

The authors declare no conflict of interest.

### Additional information

No additional information is available for this paper.
